# Management of multicausal iatrogenic bile duct injuries with biliary fistula: Twenty-year experience in a tertiary center

**DOI:** 10.1055/a-2788-3182

**Published:** 2026-02-23

**Authors:** Victor Garbay, Jean-Philippe Ratone, Cristophe Zemmour, Solene Hoibian, Yanis Dahel, Anais Palen, Jonathan Garnier, Jacques Ewald, Olivier Turrini, Marc Giovannini, Fabrice Caillol

**Affiliations:** 1 56181 Gastroenterology, Institut Paoli-Calmettes, Marseille, France; 2 56181 Statistics, Institut Paoli-Calmettes, Marseille, France; 3 56181 Endoscopy Unit, Institut Paoli-Calmettes, Marseille, France; 4 56181 Digestive Surgery Unit, Institut Paoli-Calmettes, Marseille, France; 5 56181 Institut Paoli-Calmettes, Marseille, France; 6 56181 UEMCO, Institut Paoli-Calmettes, Marseille, France

**Keywords:** Pancreatobiliary (ERCP/PTCD), Cholangioscopy, Endoscopic ultrasonography, Biliary tract, Intervention EUS, Laparoscopy

## Abstract

**Background and study aims:**

Biliary surgery is a common procedure, especially cholecystectomy (CCT). Its main adverse event (AE) is biliary duct injury (BDI). Management is poorly codified, particularly for complex BDIs not related to CCT (NONCCT-BDI). We decided to conduct a study in a tertiary center to evaluate clinical outcomes of BDI management.

**Patients and methods:**

A single-center retrospective study of patients diagnosed with a BDI between March 2002 and June 2022 was performed. The primary endpoint was the overall success rate for BDI management. Secondary endpoints were outcomes of BDI related to CCT (CCT-BDI) and non-CCT-BDI according to BDI location, need for a combination of procedures, and AEs.

**Results:**

Sixty-four patients were included. The overall success rate was 91.8%. Endoscopic retrograde cholangiopancreatography (ERCP) alone was efficient in 97.4% of patients. Endoscopy was key to successful treatment in 69% of patients. Forty-five percent of cases were non-CCT-BDI and the treatment success rate was 88.9%. The treatment success rate was significantly higher for Strasberg A BDIs ( *P* = 0.0337).

**Conclusions:**

ERCP remains the best and least invasive treatment for hilar injuries, as evidenced by a high success rate. Management of NON-CCT-BDIs should be modeled after that of CCT-BDIs. Owing to the need for a combination of treatments, complex hilar injuries must be managed in expert centers.

## Introduction

Cholecystectomy is one of the most commonly performed surgeries worldwide, with 750,000 cholecystectomies performed each year in the United States, more than 85% of which are performed laparoscopically 1 2 . Rates of morbidity and mortality related to laparoscopic cholecystectomy are lower than those of open cholecystectomy, leading to its adoption as the gold standard. The main disadvantage of laparoscopic cholecystectomy is the higher rate of biliary duct injury related to cholecystectomy (CCT-BDI), and the rate of biliary tract injury varies between 0.4% and 1.5% for laparoscopy and between 0.2% and 0.3% for open surgery 2 3 . Bilio-pancreatic surgery, particularly oncological surgery, also presents a nonnegligible risk of biliary injury. There are few studies on management of BDIs not caused by cholecystectomy (non-CCT-BDIs), so these injuries are usually managed in the same way as CCT-BDIs are. A biliary injury is a morbidity with a mortality rate of up to 8.8% 4 . Biliary tract injuries are also considered a morbidity, with progression to biliary stenosis complicating management, and have a significant impact on patients' quality of life 5 6 7 8 . Biliary tract injuries are managed via therapeutic abstention, percutaneous drainage, endoscopy and different surgical repair methods. We wanted to report and assess our practices in managing non-CCT-BDIs surgically and endoscopically in our tertiary center. The endpoint of this study was to determine the overall success rate of biliary tract injury management. The secondary endpoints were to determine success rates of CCT-BDI and nonCCT-BDI treatments, assess success rates of each technique, assess the need for a combination of treatments, assess reliability of magnetic resonance imaging (MRI), assess adverse events (AEs), assess the impact of delayed injury diagnosis, and predict the prognosis of biliary tract injuries at different locations.

## Patients and methods

### Study design and data collection

**Fig. 1:**
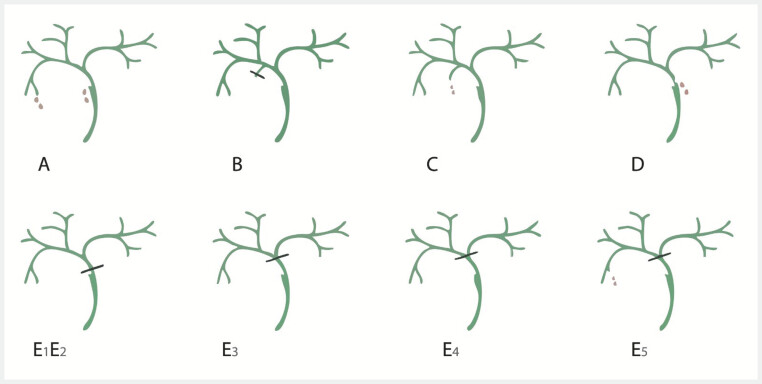
Strasberg’s classification.

A single-center retrospective study was performed at our tertiary center (Paoli-Calmettes Institute, Marseille, France) between March 2002 and June 2022. All patients were identified via the fulltext software ConSoRe. ConSoRe is a new generation of Big Data health software developed by Unicancer, one of Europe’s largest cancer research organizations. ConSoRe employs artificial intelligence based on machine learning and natural language processing. All data were collected from patient electronic medical records, which contained information on medical consultations, medical observations, and endoscopic percutaneous and surgical procedures; patients were contacted via telephone in cases of missing information. We included all patients over 18 years of age who underwent management of a postoperative biliary tract injury with a biliary fistula at the Paoli-Calmettes Institute. Patients with biliary stricture without fistula, postradial, or anastomotic stenosis, or tumor stenosis were excluded. We recorded different locations of the biliary injuries according to the Strasberg classification, which is the recommended classification for biliary injuries 9 10 . According to the Strasberg classification, E-type lesions are defined as hilar lesions associated with biliary strictures. To simplify classification of our patients, we chose to classify patients with a hilar fistula in the Strasberg E category by analogy. Our modified Strasberg classification is shown in Fig. 1 . We also recorded time to diagnosis and management, different biliary repair techniques used and their overall success rates. The primary endpoint was long-term outcome of iatrogenic biliary injury management. We defined therapeutic success as no dependency on endoscopic or percutaneous drainage (defined as the need for biliary drainage more than 1 year after completion of treatment), no need for revision surgery, no development of secondary biliary cirrhosis, no need for liver transplantation, and no biliary injury-related death. Secondary endpoints were success rates of CCT-BDI and non-CCT-BDI management, success rate for each technique used, prognosis in relation to anatomical level of the injury, and impact of time to diagnosis before management. We also wanted to determine the AE rate for each technique (complications were categorized according to the Clavien–Dindo classification), the concordance between cholangio-MRI and cholangiography in terms of biliary injury location, and the location of the biliary injury on endoscopic ultrasound (EUS). We also intended to identify factors predicting successful management 11 .

### Statistical methodology

Statistical analyses were performed using SAS 9.4 software. Categorical variable are described as numbers (percentages) and continuous variables are described as medians [min-max] and means (standard deviations). The overall success rate was calculated on the basis of the total number of evaluable patients, i.e., those who were not lost to follow-up or died during the study or follow-up period, and the exact two-sided 90% confidence interval (CI) was estimated. Associations with the following criteria were assessed via Fisher’s exact tests: operative repair technique, cholecystectomy placement, severe comorbidity, smoking status, and injury type (cholecystectomy vs. surgery/radiofrequency vs. endoscopic injury).

### Ethical considerations

Approval was obtained from the Institutional Review Board (IRB) and the local medical ethics committee. The IRB number assigned to this study was BDI2022-IPC 2022–05.

## Results

### Characteristics of patients and biliary duct injuries

**Fig. 2:**
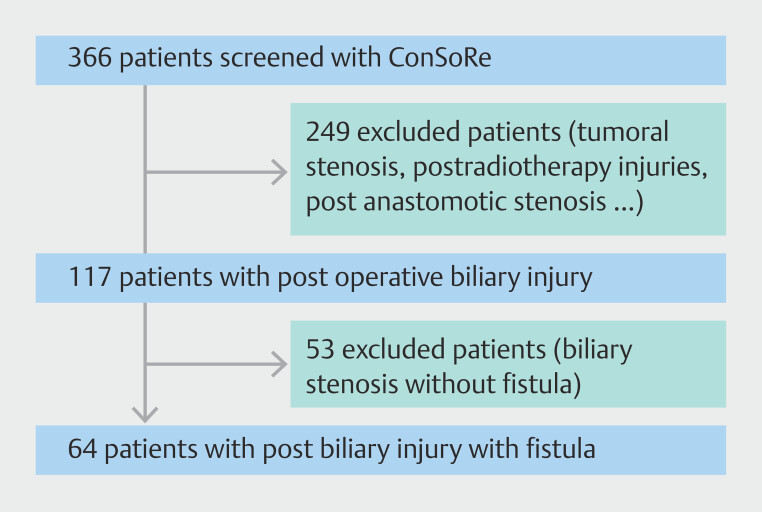
Flow chart.

Using ConSoRe software, we identified 366 patients after searching using the terms "biliary," "injury," and "fistula". Among these 366 patients, 249 were excluded because they did not have a postoperative biliary injury. Finally, among the 117 patients with a biliary injury, 53 were excluded because they had a biliary stricture without a fistula. Sixty-four patients (32 men and 32 women) were included between March 2002 and June 2022 ( Fig. 2 ), and mean age was 64.94 ± 17.36 years [18–90]. Sixty-one percent of patients had at least one serious comorbidity. Thirty-five patients (55%) had a biliary injury secondary to cholecystectomy. A total of 24 of 64 BDIs (37%) were discovered within 72 hours (including 19 intraoperatively). A total of 30 of 64 (47%) were found after 72 hours, seven of which were found after 7 days. A total of 15 of 64 patients (23%) were diagnosed during discovery of a bilioma and 14 of 64 (22%) were diagnosed during treatment of biliary peritonitis. Some patients were diagnosed after a leak in the surgical drain was discovered (13/64) or developing jaundice (3/64). Thirty patients (47%) had Strasberg A injuries, 11 patients (17%) had Strasberg C injuries, 14 (22%) had Strasberg D injuries, and nine patients (14%) had Strasberg E injuries. With respect to the Strasberg grade, 44 of 64 patients (69%) had a grade 1 injury, 19 of 64 (30%) had a grade 2 injury, and one of 64 (1%) had a grade 3 injury. A total of 18 of 64 patients (28%) in our study underwent cholangio-MRI. Every MRI performed was using 1.5T equipment. Characteristics of patients and BDIs are summarized in Table 1 .

**Table:** **Table 1** Patient and biliary duct injury characteristics.

	**Total**	**CCT-BDI**	**Non-CCT-BDI**
**Number of patients**	n = 64	n = 35 (55%)	n = 29 (45%)
**Sex**			
Male	n = 32 (50%)	n = 18 (51%)	n = 14 (48%)
Female	n = 32 (50%)	n = 17 (49%)	n = 15 (52%)
**Mean age**	64.94 +/- 17.36 years [18–90]	65.7 +/- 18.4 years [18–89]	64 +/- 16.3 years [19–90]
**Patients with ≥ 1 major comorbidity**	n = 39 (61%)	n = 18 (51.4%)	n = 20 (72.4%)
**Type of biliary injury**			
Post cholecystectomy	n = 35 (55%)		
Post carcinological hepatectomy	n = 17 (26.5%)		
Post non carcinological hepatectomy	n = 1 (1.5%)		
Post endoscopy	n = 10 (15.5%)		
Post radiofrequency	n = 1 (1.5%)		
**Time to detection**			
intraoperative	n = 20 (31%)	n = 15 (42.9%)	n = 5 (17.2%)
< 72h	n = 14 (22%)	n = 9 (25.7%)	n = 5 (17.2%)
> 72 h to < 7 days	n = 9 (14%)	n = 5 (4.3%)	n = 4 (13.8%)
> 7 days	n = 21 (33%)	n = 6 (17.1%)	n = 15 (51.7%)
**Mode of detection**			
Perioperative	n = 19 (30%)	n = 14 (40.0%)	n = 5 (17.2%)
Bilioma	n = 15 (23%)	n = 3 (8.6%)	n = 12 (41.4%)
Biliary peritonitis	n = 14 (22%)	n = 5 (14.3%)	n = 9 (31.0%)
Biliary leak in the surgical drain	n = 13 (20%)	n = 10 (28.6%)	n = 3 (10.3%)
Jaundice	n = 3 (5%)	n = 2 (5.7%)	n = 1 (3.4%)
**Location of biliary injury**			
Strasberg A	n = 30 (47%)	n = 15 (42.9%)	n = 15 (51.7%)
Strasberg B	n = 0 (0%)	n = 0 (0%)	n = 0 (0%)
Strasberg C	n = 11 (17%)	n = 8 (22.9%)	n = 3 (10.3%)
Strasberg D	n = 14 (22%)	n = 6 (17.1%)	n = 8 (27.6%)
Strasberg E1	n = 4 (6%)	n = 3 (8.6%)	n = 1 (3.4%)
Strasberg E2	n = 1 (1.5%)	n = 0 (0%)	n = 1 (3.4%)
Strasberg E3	n = 3 (5%)	n = 3 (8.6%)	n = 0 (0%)
Strasberg E4	n = 1 (1.5%)	n = 0 (0%)	n = 1 (3.4%)
**Performing a cholangio-MRI**			
Yes	n = 18 (28%)	n = 13 (37.1%)	n = 5 (17.2%)
before treatment	n = 10 (15.6%)	n = 7 (20%)	n = 3 (10.3%)
during treatment	n = 8 (12.4%)	n = 6 (17%)	n = 2 (6.9%)
No	n = 46 (72%)	n = 22 (62.9%)	n = 24 (82.8%)
BDI, biliary duct injury; CCT, cholecystectomy; MRI, magnetic resonance imaging.			

### Procedure characteristics

A total of 40 of 64 patients (63%) were managed with endoscopic retrograde cholangiopancreatography (ERCP) first and 52 of 64 patients (81%) underwent at least one ERCP procedure. Among these patients, 42 of 52 (80%) underwent placement of one or more plastic stents, three of 52 (6%) underwent placement of only fully covered self-expandable metal stents (FC-SEMSs), six of 52 (12%) underwent placement of both plastic stents and FC-SEMSs, and one of 52 (2%) underwent only biliary sphincterotomy. Every patient that had a stent placement (plastic or metallic) had transpapillary stenting. One patient (2%) underwent EUS hepaticogastrostomy. Five patients (8%) underwent combined percutaneous treatment and ERCP, and eight patients (13%) underwent surgical management. Eight patients underwent combined surgical and ERCP treatment (13%). Two patients (3%) were managed without an interventional procedure (simple monitoring). Procedure characteristics and injuries are shown in Table 2 .

**Table:** **Table 2** Procedure characteristics, success rate, and adverse events rates for each technique.

**ERCP first management**	n = 40
Therapeutic success	n = 38/39 (97.4%)
Therapeutic failure	n = 1
No evaluation possible	n = 2
Adverse events	n = 4 (6%)
Clavien Dindo I	n = 1
Clavien Dindo IIIb	n = 3
**EUS management**	n = 1
Therapeutic success	n = 0 (0%)
Therapeutic failure	n = 1
Adverse events	n = 0 (0%)
**Endoscopic and percutaneous management**	n = 5
Therapeutic success	n = 5/5 (100%)
Therapeutic failure	n = 0
Adverse events	n = 0 (0%)
**Exclusive surgical management**	n= 8
**Therapeutic success**	n = 6/8 (75%)
**Surgical suture**	n = 1
Therapeutic success	n = 1
Adverse events	n = 0 (0%)
Hepaticojejunal anastomosis	n = 7
Therapeutic success	n = 5/7 (71.5%)
Adverse events	n = 2/7 (29%)
Clavien Dindo IIIB	n = 2
**Combined surgical and ERCP management**	n = 8
Therapeutic success	n = 6/6 (100%)
Adverse events	n = 0
**Therapeutic abstention**	n = 2
Therapeutic success	n = 2
ERCP, endoscopic retrograde cholangiopancreatography; EUS, endoscopic ultrasound.	

### Primary endpoint: overall success rate

We report an overall success rate of 91.8% (83.5%-96.7%) Treatment was successful in 56 of 61 evaluable patients. The failure rate was reported for five patients (8.2%): two patients died because of biliary injury and three patients were dependent on endoscopic drainage. Three patients could not be evaluated for success: two patients were lost to follow-up and one patient died during treatment from a pathology unrelated to the biliary injury. All the details concerning the primary endpoint are shown in Table 3 .

**Table:** **Table 3** Primary endpoint.

**Overall success rate**	**n = 57/61 (93.4%)**
**Failure rate**	n = 5 (8%)
Death in the aftermath of the biliary injury	n = 2 (3%)
Dependence on endoscopic drainage	n = 3 (5%)
**Patients not evaluable for success**	n = 3 (5%)
Lost to follow-up	n = 2 (3%)
Death during treatment	n =1 (1%)

### Secondary endpoints

#### Success rates for CCT-BDI and non-CCT-BDI management

The success rate was 94.3% (n = 33/35) in the CCT-BDI subgroup and 88.9% (n = 24/27) in the non-CCT-BDI subgroup. Characteristics of the two subgroups were similar in terms of Strasberg type and treatment type ( Table 4 ).

**Table:** **Table 4** Patient characteristics and success rates in the CCT-BDI and non-CCT-BDI subgroups.

	**CCT-BDI n = 35 (55%)**	**Non-CCT-BDI n = 29 (45%)**
**Strasberg classification**		
A	15	15
C	8	3
D	6	8
E1	3	1
E2	0	1
E3	3	0
E4	0	1
E5	0	0
**Treatment of BDI**		
Endoscopy	22 (61%)	18 (62%)
EUS	0 (0%)	1 (3%)
Surgery	6 (17%)	2 (7%)
Suture	1	0
Anastomosis	4	2
Surgery + endoscopy	5 (14%)	3 (11%)
Percutaneous + endoscopy	3 (8%)	2 (7%)
Abstention	0	2 (7%)
**Success**		
Yes	32 (94.1%)	24 (88.9%)
NA	1	1
No	2 (5.9%)	3 (11.1%)
BDI, biliary duct injury; CCT, cholecystectomy.		

#### Success rate according to location of biliary injury

We report a success rate of 96.7% for Strasberg A bile duct injuries, 100% for patients in the Strasberg C, E1 and E2 groups, 92.8% for Strasberg D injuries, and 50% for those in the Strasberg E3 group. Note that the only patients with a Strasberg E4 injury also experienced failure. After univariate analysis, the success rate for treatment of Strasberg type A biliary injuries was significantly higher than that for treatment for biliary injuries at other locations ( *P* = 0.0337) ( Table 5 ). Every type of treatment according to different locations of bile duct injuries is shown in Table 6 .

**Table:** **Table 5** Univariate analysis of treatment success.

**Parameters**	***P* value (Wilcoxon or Fisher) **
Strasberg classification (A vs other)	0.0337
Major comorbidity	1
Injury management time	0.0652
Intraoperative repair	1
Type of biliary injury	0.5428
Use of MRI	1
MRI, magnetic resonance imaging.	

**Table:** **Table 6** Procedure characteristics, success rate, and adverse events rates for each technique.

**Treatment**	**Strasberg A (n=30)**	**Strasberg C (n=11)**	**Strasberg D (n = 14)**	**Strasberg E1 (n = 4)**	**Strasberg E2 (n = 1)**	**Strasberg E3 (n=3)**	**Strasberg E4 (n=1)**	**Total**	**Success rate**
**Endoscopy**									
Total								**40**	37/38 (97,4%)
BDI-CCT	12 (S=12)	7 (S=7)	2 (S=2)			1 (NA =1)		**22**	21/21 (100%)
BDI-non-CCT	9 (S=8, F=1)	3 (S=3)	5 (S=4, NA =1)	1 (S=1)				**18**	16/17 (94%)
**EUS**									
Total								**1**	0/1 (0%)
BDI-CCT									
BDI-non-CCT								**1**	0/1 (0%)
**Surgery**								**8**	6/8 (75%)
**Suture**									
Total		1 (S=1)						**1**	1/1 (100%)
BDI-CCT		1 (S=1)						**1**	1/1 (100%)
BDI-non-CCT									
**Anastomosis**									
Total								**7**	5/7 (71,4%)
BDI-CCT								**5**	3/5 (60%)
BDI-non-CCT								**2**	2/2 (100%)
**Surgery + endoscopy**									
Total								**8**	6/6 (100%)
BDI-CCT	2 (S=2)		1 (S=1)	1 (S=1)		1 (NA =1)		**5**	4/4 (100%)
BDI-non-CCT	2 (S=2)		1 (NA =1)					**3**	2/2 (100%)
**Percutaneous + endoscopy**									
Total								**5**	5/5 (100%)
BDI-CCT			1 (S=1)	2 (S=2)				**3**	3/3 (100%)
BDI-non-CCT	1 (S=1)		1(S=1)					**2**	2/2 (100%)
**Abstention**									
Total								**2**	2/2 (100%)
BDI-CCT									
BDI-non-CCT								**2**	2/2 (100%)
**Total**	**30**	**11**	**14**	**4**	**1**	**3**	**1**	64	
**Success rate**	29/30 (96,7%)	11/11 (100%)	12/13 (92,8%)	4/4 (100%)	1/1 (100%)	1/2 (50%)	0/1 (0%)	56/61 (91.8%)	
BDI, biliary duct injury; CCT, cholecystectomy; EUS, endoscopic ultrasound; F, failure; NA, not applicable; S, success.									

### Adverse events

We report an endoscopic AE rate of 5% (2 AEs were noted in the CCT-BDI subgroup and 2 were noted in the non-CCT-BDI subgroup). We did not report any complications in patients who received percutaneous or EUS management. We did not report any immediate complications of surgical management, but among the six patients who benefited from hepaticojejunal anastomosis, two presented with anastomotic stenosis requiring management by endoscopic drainage, i.e., a stenosis rate of 33% ( Table 2 ).

### Success rate according to management delay

Among the 19 patients managed intraoperatively, 90% exhibited therapeutic success, with a success rate of 93% for those managed within 3 days, 89% for those managed within the first 7 days, and 81% for those managed beyond 7 days ( *P* = 0.0652) ( Table 5 ).

### Factors for overall therapeutic success

According to univariate analysis, overall success of treatment was significant for only Strasberg A biliary injuries ( *P* = 0.0337). There was no significant association with the other factors studied (presence of an associated heavy comorbidity, type of biliary injury, immediate intraoperative repair, and delayed diagnosis) ( Table 5 ).

### Success rate according to performance of cholangio-MRI

One hundred percent of patients who underwent cholangio-MRI before treatment experienced therapeutic success, whereas 75% of patients who underwent cholangio-MRI during treatment experienced therapeutic success. A total of 89% of patients who underwent MRI experienced therapeutic success. There was no significant difference in therapeutic success between the groups that underwent or did not undergo cholangio-MRI ( *P* = 1). The concordance rate for presence and location of biliary injury between cholangio-MRI and cholangiography was 89%. Notably, the two patients for whom a discrepancy in biliary injury identification was observed between cholangiography and bili-MRI experienced clinical success ( Table 5 ).

### Use of therapeutic EUS

Three patients underwent EUS, two patients underwent transmural drainage of a bilioma and subsequent endoscopic management, both of which were successful, and one patient underwent EUS for bile duct access and subsequent injury management.

## Discussion

Management of nontraumatic biliary injuries requires a multidisciplinary approach because they are poorly coded. The aim of our study was to evaluate success rates for different techniques to propose the optimal management strategy for each type of biliary injury. We chose to exclude patients with stenosing biliary injuries without fistulas to clarify management and homogenize the study population. In our study, although retrospective and monocentric, we grouped the 64 patients according to management strategy and compared several different management methods. Our study is also the largest study to date. Our study, which was conducted in a tertiary center, revealed an overall success rate of 91.8%.

One of the strengths of this study is the high number of non-CCT-BDI patients included (n =29/64, 45%). Studies on non-CCT-BDI are rare, and their management is complex because they are less codified than CCT-BDI 12 . These non-CCT-BDIs are more often complex and difficult to manage. In our study, the CCT-BDIs and non-CCT-BDIs benefited from personalized management even though treatment outcomes were similar. These results lead us to believe that treatment for non-CCT-BDIs could have equivalent outcomes if the location of the BDI is well identified. Although ERCP remains the most commonly performed treatment to date, it is not the only treatment.

Among the different treatment options, ERCP allows accurate identification of the biliary injury location and, therefore, optimal management of the injury and any associated stricture or choledocholithiasis. ERCP also has a low complication rate (5% in our study). The therapeutic success rate for ERCP in the cases mentioned above varies between 87% and 100%, which is consistent with the 97.4% success rate reported in our study 13 14 15 16 . In our study, most patients received endoscopic treatment (65% as primary treatment and 81% all together). However, the rate of ERCP and its success can be explained by the high rate of nonsevere Strasberg biliary tract injuries, which are easier to manage endoscopically. With respect to endoscopic treatment techniques, different treatment options have been compared. The European Society of Gastrointestinal Endoscopy recommends use of plastic stents for 4 to 8 weeks in patients with biliary injuries; however, complete sections of the main bile duct should not be blocked 17 . Use of FC-SEMSs can be justified in cases of refractory injuries 18 . These recommendations were recognized globally because most patients benefited from placement of one or more plastic stents (81%).

Use of EUS to access the bile ducts and manage benign situations is a new therapeutic option 19 . In our study, one patient underwent EUS (hepaticogastrostomy) for management of a complex hilar BDI. This management was concluded to be a failure due to patient dependance on subsequent biliary drainage but was beneficial for quality of life (absence of skin material). Transduodenal drainage of the right intrahepatic bile ducts for management of CCT-BDI was also described 20 . EUS seems to be beneficial in management of biliary injuries, bilioma drainage, and accessing bile ducts; additional studies are necessary to confirm the importance of EUS in management of biliary injuries.

Concerning surgical repair of biliary tract injuries, the World Society of Emergency Surgery (WSES) published recommendations for management of biliary tract injuries in 2020 9 . Concerning intraoperative management of biliary injuries, the WSES recommends placement of a T-tube with or without injury repair in the case of an injury that is considered minor (Strasberg A-D and E2) and it also recommends performing a Roux-en-Y hepaticojejunostomy in case of a major injury and delaying management of complex injuries (vasculobiliary) without attempting perioperative management. In our series, all the preoperative suturing procedures for bile duct injuries were successful. Hepaticojejunostomy was successful in half the patients if it was performed at time of discovery of the BDI and in two-thirds of patients if it was performed at a later date. This rate of anastomotic stricture is higher than that reported in previous studies, which revealed a rate varying between 10% and 20% 21 . This can be explained by the fact that all of these patients did not undergo surgery at a tertiary center but were referred to us after.

Therefore, our series confirms that ERCP must be the first-line treatment for extrahepatic injury and that Roux-en-Y hepaticojejunal anastomosis must be delayed and performed in expert surgical centers. Simple surgical drainage, and then referral of the patient to an expert center specializing in endoscopic, percutaneous, and surgical treatment of biliary injuries seem to be the best option, especially for patients with complex BDIs.

In our study, five patients underwent minimally invasive combined management (endoscopic and percutaneous), with 100% therapeutic success. A comparison with the ERCP success rate is not possible because combined minimally invasive management is performed more often for complex BDIs. However, it is clear that ERCP would not have been sufficient in these cases. Owing their high success rates, percutaneous approaches are traditional second-line management approaches when ERCP is not sufficient. In the Belgian study by Lorenzo et al., 16 patients with a complex biliary injury received mixed management via transpapillary and transfistula access 22 . The reported success rate was 67%. Although comparisons are difficult, these results prove that combined management is efficient for complex biliary injuries, with a high success rate in expert centers.

In our study, the success rate for therapeutic abstention was 100% in two patients with a low-flow fistula (distal intrahepatic BDI). This finding is in accordance with a 2019 review of 10 studies in which researchers opted for simple follow-up without intervention, revealing a success rate of 91% 23 . However, teams must consider this option carefully and commit to closely following these patients.

We have summarized all treatments according to BDI location in Table 6 . This table highlights several key points. The success rate decreases as complexity of the biliary injury increases, regardless of treatment modality. This trend is confirmed in Table 5 , where the Strasberg A group shows a significantly higher success rate than the other groups (96.7%, *P* = 0.0337). To clarify management of bile duct injuries, we can separate different locations of BDI by grouping bile duct injuries distant from the hilum (Strasberg A, C, and D) vs. bile duct injuries in the hilum (Strasberg E). The group of patients with BDI distant from the hilum (Strasberg A, C, and D) included 55 patients with a success rate of 96% (n = 52/54). In this group, 70% of patients (n = 38/55) benefited from exclusive endoscopic management with a success rate of 97% (n = 37/38). The group of patients with hilar BDI (Strasberg E) included nine patients with a success rate of 75% (n = 6/8). For hilar BDIs management was heterogeneous: two by ERCP alone, one by EUS, two by percutaneous treatment plus endoscopy, two by surgery alone, and two by surgery plus endoscopy. These data confirm that endoscopy remains the best treatment for bile duct injuries distant from the hilum due to its high success rate, accessibility, and low morbidity. On the other hand, hilar BDIs are challenging to treat due to numerous modalities of treatments and lower success rates. Their management must be collaborative, with access to all possible options, which therefore justifies referral to a tertiary care center. Based on this study, it seems difficult to clearly define the type of treatment required for these BDIs. This can be explained in part by one of the limitations of this study, which is that although the overall number of biliary injuries is high compared with other studies, the many subclasses result in a small number of patients per treatment category or BDI location. A study with a larger sample size would be valuable to better define optimal treatment for each BDI location.

## Conclusions

In conclusion, our study, which was conducted at a tertiary center, revealed an overall success rate of 97.4% for endoscopic management that should be the standard treatment for BDI distant from the hilum. Endoscopic management of BDIs unrelated to cholecystectomy appear to benefit from the same management strategy used for BDIs related to cholecystectomy with the same outcome. Complex hilar injuries should be identified early, surgical management should be delayed, and patients should be referred to tertiary centers for management by expert multidisciplinary teams.

## Notes

Correction **Management of multicausal iatrogenic bile duct injuries with biliary fistula: Twenty-year experience in a tertiary center** Victor Garbay, Jean-Philippe Ratone, Cristophe Zemmour et al. Endoscopy International Open 2026; 14: a27883182. DOI: 10.1055/a-2788-3182 In the above-mentioned article the last author's name was corrected. This was corrected in the online version on 23.03.2026.
