# Correction: Management of multicausal iatrogenic bile duct injuries with biliary fistula: Twenty-year experience in a tertiary center

**DOI:** 10.1055/a-2837-2157

**Published:** 2026-03-25

**Authors:** Victor Garbay, Jean-Philippe Ratone, Cristophe Zemmour, Solene Hoibian, Yanis Dahel, Anais Palen, Jonathan Garnier, Jacques Ewald, Olivier Turrini, Marc Giovannini, Fabrice Caillol

**Affiliations:** 1 56181 Gastroenterology, Institut Paoli-Calmettes, Marseille, France; 2 56181 Statistics, Institut Paoli-Calmettes, Marseille, France; 3 56181 Endoscopy Unit, Institut Paoli-Calmettes, Marseille, France; 4 56181 Digestive Surgery Unit, Institut Paoli-Calmettes, Marseille, France; 5 56181 Institut Paoli-Calmettes, Marseille, France; 6 56181 UEMCO, Institut Paoli-Calmettes, Marseille, France



In the above-mentioned article the last author's name was corrected. This was corrected in the online version on 23.03.2026.

